# Cancer Genetics and Clinical Research

**DOI:** 10.3390/jpm12101649

**Published:** 2022-10-04

**Authors:** Sarah Allegra

**Affiliations:** Laboratory of Clinical Pharmacology “Franco Ghezzo”, Department of Clinical and Biological Sciences, University of Turin, San Luigi Gonzaga Hospital, 10043 Orbassano, Italy; sarah.allegra@gmail.com

Understanding how complex diseases as well as cancers arise is one of the great challenges of modern medicine. Cancer is defined as a genetic disease because it is caused by mutations in genes which control cell grow and proliferation mechanisms [1]. Yet, cancer is hereditary only when there is a specific oncogenic mutation inherited from previous generations. However, a lot of genes identified in familiar cancer syndromes have a role also in sporadic type of cancers. In addition, not only genetics control this malignancy, but also exposome and metabolome play a role in determining and sustaining cancer [2].

In recent years, significant advances have been made in understanding tumor genetic alterations, especially thanks to the advent of next generation sequencing (NGS) technologies, which have enormously accelerated discoveries and increased the production of data. The main innovation consisted precisely in moving from targeted sequencing techniques of a few genes in a few samples to the possibility of studying thousands of genes simultaneously, to then arrive at the massive sequencing of entire exomes and, if desired, the entire genome. The first revolution occurred when, alongside the classic investigations of molecular biology and cytogenetics, such as the polymerase chain reaction (PCR) and fluorescent in situ hybridization (FISH), capable of analyzing only one gene at a time, the microarray technologies expression and single nucleotide polymorphism (SNP) arrays were able to quantify the expression profile of all known genes expressed by a cell, and to identify all the amplification or deletion regions of the genome [3,4]. With microarrays, it was possible, for the first time, to map all the genetic alterations of cancer at a high resolution, without a prior knowledge of the candidate gene.

However, these technologies do not allow us to see alterations such as mutations, inversions, and fusion transcripts. On the other hand, NGS sequencing is able to see every type of alteration present from mutations to copy number variations, and to detect fusion transcripts and quantify the level of gene expression. Mutations were initially detected by Sanger Sequencing, which involved the amplification of a region of interest and then, through the incorporation of labeled dideoxynucleotides, to read the nucleotide base sequence on a capillary sequencer. This technique represents the first generation method for sequencing [5]. The second generation or next-generation techniques were then developed which made it possible to sequence thousands of different regions in parallel, enormously increasing the amount of data which was produced and analyzed in a short space of time, up to the possibility of sequencing the entire human genome in a single run [6]. The sample is fragmented and amplified, obtaining a DNA *library* which will then be immobilized on a solid support or *beads* in order to sequence all the fragments in parallel [7]. In the sequencing process, the incorporation signals of the different nucleotides are converted into *reads*. This procedure, called *base-calling*, detects a series of parameters, such as the intensity of the signal, background noise, and the presence of non-specific signals, through specific algorithms. Eventually, it generates the nucleotide sequences and assigns scores to each base (*quality scores*). The latter are related to the probability of error and are a useful tool for eliminating bases or excluding *reads* that show a low quality. In this way, the accuracy of the alignment is improved [8]. The *reads* are then aligned on the reference genome and then, through special bioinformatics pipelines, the data are analyzed and all the alterations of interest are identified. In clinical practice, NGS helps clinicians to investigate germline and somatic mutation, targeted panels, whole-exome sequencing (WES), and/or whole-genome sequencing (WGS), with a single test [3].

WGS allow us to analyze the genome in its entirety, identifying structural variants and single nucleotide variants (SNVs), both in the coding and in the intergenic regions. To obtain the genomic DNA *libraries* to be sequenced, the DNA of interest is fragmented and amplified, joining adaptive sequences at the ends of each fragment that will allow it to be attached to the sequencing *flowcell*. The main limitations of this technique are represented by the enormous size and complexity of the data to be analyzed and the considerable costs required. For these reasons, WES is much more frequently used, capable of analyzing only the coding regions of the genome, i.e., the exome, thus reducing the complexity and size of the data, and therefore the costs [9]. To make WES, genomic DNA *libraries* are hybridized to specific probes in order to enrich only the coding regions. From the WES data, it is therefore possible to identify all the SNVs and INDELs in the coding regions involved in splicing; moreover, it is possible to estimate the copy number, using the data covering the *reads* in the single sequenced regions. Eventually, whole-transcriptome sequencing (RNA-seq) allows the entire transcriptome to be sequenced from mRNA *libraries*, which are also fragmented and amplified by joining adaptive sequences for binding to the *flowcell*. It allows us to quantify the gene expression profile, to identify the presence of rearrangements producing fusion genes, and to evaluate alternative splicing. It is also possible, with specific analysis pipelines, to identify SNVs and INDELs. The main limitations of transcriptome analysis are represented by the inability to identify the presence of variants in the regions not expressed in the sample. These sequencing systems are today the optimal tools for the characterization of the alterations present in the various tumor types, allowing comprehensive analyses at relatively low costs and in a short time frame [10]. 

CRISPR/Cas9 is a genome editing method based on the observation of a prokaryotic defense system and based on the presence of an RNA guide, associated with a nuclease capable of recognizing a specific sequence. The CRISPR/Cas system, used for the modification of the genome for therapeutic or industrial purposes, is not an artificial system but has a biological origin as it has been found in various prokaryotic species, where it performs the function of specifically recognizing the exogenous DNA and destroying it. The CRISPR loci consists of a series of non-contiguous repeated sequences separated by variable sequences with a length of about 30 bp, called *spacer*; the associated Cas genes are located in proximity to the CRISPR loci and code for a wide variety of proteins with different functions. First observed in 1987 during the study of the *iap* enzyme in Escherichia coli [11], its function remained unknown until 2005, when it was noted that the *spacer* sequences possessed sequence homology with DNA elements of external origin [12]. The CRISPR/Cas system is an adaptive immune system, thanks to which resistance to mobile genetic elements is acquired. Its operation can be summarized in three main steps: (1) the adaptation step: “new” spacers of exogenous nucleic acids belonging to the virus, which infects the cell, are digested by bacterial nucleases and acquired in the CRISPR locus; (2) crRNA (crispr RNA) biogenesis: through transcription and processing by an endoribonuclease of the CRISPR array in small interfering crRNAs; and (3) the targeting step: the crRNA together with the tracrRNA (trans-activating crRNA) guides the Cas9 nuclease on the complementary sequences to carry out the cut of the exogenous nucleic acids. CRISPR/Cas9 gene editing techniques are widely used to manipulate cancer cells genome, as a new option for treatment, and for drug resistance overcome [13].

Though we are not made of just genes. More than half of the risk of developing a certain disease is due to environmental factors [14], hence the need to develop a new analysis paradigm that includes the study of the exposome: a detailed map of all the environmental components to which an individual is exposed to throughout his life, and how those exposures affect their health. Yet the exposome analysis should also take into account what happens inside our body: oxidative stress phenomena or inflammatory processes contribute every day to create an extremely dynamic internal environment. Well, even the by-products of these biological phenomena, as well as their interaction with other substances, cannot be overlooked in the analysis of the exposome. The same influence should be considered in the study of environmental factors: only through the analysis of all the environmental factors to which an individual is exposed, the information about the actual role that they play in genes interaction and influencing health can be gained [15].

Metabolomics is the study of all metabolites in a cell or in an organism [16]. Small changes in the concentrations or activities of enzymes can cause large changes in metabolite levels. These alterations could be used to classify different types of cancer and identify new prognostic and predictive markers. The concept of a metabolic profile dates back to 1927 and refers to the characteristic pattern of the metabolites of each individual, present in body fluids such as urine and saliva [17]. In 2007, the Human Metabolome Project, led by a Canadian team, completed a first draft of the human metabolome which, in interactions, also includes drugs and food [18]. The Human Metabolome Database (www.hmdb.ca, accessed on 19 September 2022) contains information on over 40,000 metabolites that have been analyzed using standardized procedures; the information is gathered through a periodic review of the scientific literature and is constantly updated [19]. The chromatography techniques, alone or in combination with mass spectrometry, nuclear magnetic resonance for spectroscopic analysis, and capillary electrophoresis are the most widely used methods for metabolomics [20,21]. Functional genomics uses the results of metabolomics to understand the impact of a genetic mutation on a cellular metabolism.

Metabologenomics aims to integrate metabolomics and genomics data through the correlation between similar synthesis pathways, identified through analogies between enzymes and coding genes, to identify unknown metabolites. The metabolomics techniques have allowed and will allow us to deepen the pathophysiology of various tumors and to plan diagnostic strategies that will be of common use in clinical practice over the next few years. The future vision offered by metabolomics provides for a diagnostic simplification of neoplastic pathologies and a more accurate identification of malignant lesions, from onset to relapse. In addition, from a multi-analytical assessment point of view, the combination of metabolomics and other -omics techniques may provide yet unexplored notions of tumor pathophysiology [2,22,23].

Clinical researches have the aim of studying the interplay between a multi-omics view of cancer and genomic, exposomic, and metabolomic views, evaluating treatment options, utilizing mechanisms of prevention and diagnosis, screening tools, and patient quality of life evaluation to gain a precision in medicine. Tailored approaches in cancer research should be a critical component of personalized diagnosis and treatment for each patient and condition.
